# Backstage of Eating Disorder—About the Biological Mechanisms behind the Symptoms of Anorexia Nervosa

**DOI:** 10.3390/nu12092604

**Published:** 2020-08-27

**Authors:** Kamil Skowron, Magdalena Kurnik-Łucka, Emil Dadański, Barbara Bętkowska-Korpała, Krzysztof Gil

**Affiliations:** 1 Department of Pathophysiology, Jagiellonian University Medical College, Czysta St 18, 31-121 Krakow, Poland; kamil.skowron@uj.edu.pl (K.S.); magdalena.kurnik@uj.edu.pl (M.K.-Ł.); emil.dadanski@gmail.com (E.D.); 2 Department of Psychiatry, Jagiellonian University Medical College, Institute of Medical Psychology, Jakubowskiego St 2, 30-688 Krakow, Poland; barbara.betkowska-korpala@uj.edu.pl

**Keywords:** anorexia nervosa, eating disorders, starvation, hyperactivity, neuropeptides, microbiome–gut–brain axis

## Abstract

Anorexia nervosa (AN) represents a disorder with the highest mortality rate among all psychiatric diseases, yet our understanding of its pathophysiological components continues to be fragmentary. This article reviews the current concepts regarding AN pathomechanisms that focus on the main biological aspects involving central and peripheral neurohormonal pathways, endocrine function, as well as the microbiome–gut–brain axis. It emerged from the unique complexity of constantly accumulating new discoveries, which hamper the ability to look at the disease in a more comprehensive way. The emphasis is placed on the mechanisms underlying the main symptoms and potential new directions that require further investigation in clinical settings.

## 1. Introduction

Anorexia nervosa (AN) affects primarily adolescent girls and young women, and presents with a loss of appetite, underweight, as well as endocrine alterations. In clinical settings, the ratio of adult females to males ranges from 10:1 to 20:1 [1,2]. The median age of onset for anorexia nervosa is supposed to be 18 years [3]. The lifetime prevalence of AN was found to be 0.3% among adolescents aged 13–18 years in USA [4] and 1–4% among girls aged 12–18 years in Europe [5]. Recent studies report incidence rates of 100–200/100,000 person-years in women aged 15–19 years, which is comparable to, for example, diabetes type 1 [6,7,8]. Recently, the number of hospitalized children and adolescents has increased in UK [9] and the age of onset has decreased [10].

Amenorrhea commonly occurs in AN and was a diagnostic criterion in Diagnostic and Statistical Manual of Mental Disorders (DSM)-IV [11]. DSM-V eliminated amenorrhea as a criterion because patients who menstruate but meet other criteria for AN have similar outcomes to patients who do not menstruate [12]. Thus, to fulfill the diagnostic DSM-V criteria of AN, one must restrict energy intake to induce body weight loss, exhibit a fear of gaining weight or behavior preventing weight gain or indicate a lack of understanding of the consequences of low body weight [11]. In general, two subtypes of AN can be distinguished: Restricting type (ANR), where food intake is limited, and purging type (ANBP), where self-induced vomiting or laxatives counteract food intake [13].

Food restriction and subsequent malnutrition in AN directly lead to severe multi-organ complications, such as gastrointestinal, cardiac, pulmonary, hematologic, musculoskeletal, neurologic, and dermatologic. Most of them are treatable after weight gain, effective medical interventions and psychotherapy, especially given the relatively young age of onset of AN [14]. Those complications resemble simple starvation (semistarvation) from the pathological point of view yet, underlying pathomechanisms responsible for the development of the disorder remain poorly understood. The high prevalence of fitness and thinness, the low prevalence of AN, together with a clear evidence of anorexia occurring in the past centuries, its stereotypic presentation, heritability, and developmentally specific age-of-onset, suggest rather biological accountabilities. Brain neuropeptides together with monoamine systems, especially serotoninergic and dopaminergic, are of most interest in AN, yet our understanding of the pathophysiologic role of those systems in patients is still rather limited. A disturbance of brain serotoninergic networks predates the onset of AN, and should contribute to premorbid symptoms of anxiety, inhibition, and a vulnerability for restricted eating. What is more, puberty-related steroids or other age-related changes may enhance serotonin dysregulation, as well as stress and/or cultural and societal pressures may aggravate the disease [15]. However, there is only minimal to moderate evidence that available psychiatric medications are effective [14,15,16].

According to literature, up to 40–80% of AN patients also show excessive levels of physical activity. Thus, hyperactivity, defined as rigorous physical activity combined with a compulsive need to exercise, plays a fundamental role in the development and maintenance of AN, may precede food restriction and accelerate body weight loss once food restriction has been initiated, and obviously interferes with the recovery process, and has been reported as one of the predictive factors of a higher risk of relapse after recovery [17,18,19,20,21]. The nature of this feature remains uncertain although it was already recognized and described by Gull and Lasègue in the 19th century [20,21]. Rewarding activation upon reduced energy intake through dopaminergic reinforcing pathways, hypoleptinemia and thermoregulatory compensation due to hypothermia have been hypothesized as the leading causes of hyperactivity [18,19,22,23] but genetic factors regulating activity levels should also contribute to the development of the disease [18]. Those rewarding effects, triggering euphoria and dependence, are mediated by an enhanced mesolimbic dopamine release through an activation of the hypothalamo–pituitary–adrenal axis with high blood cortisol levels due to starvation and/or hyperactivity [24]. Yet, it should be stressed that persistently increased activity levels in the presence of a negative energy balance are unique to anorexia as compared to semistarvation [17].

Thus, the aim of our review was to summarize current concepts regarding pathogenesis and pathomechanisms of AN, such as alterations of brain neurotransmission, including abnormally functioning corticolimbic circuits involved in appetite, fronto-striatal networks together with autonomic nervous system dysfunction as well as the microbiome–gut–brain axis and endocrine alterations. Original reports and review articles written in English indexed in Medline database from the last two decades (older but frequently cited publications were not ignored) were selected based on their clinical relevance, however, the process of articles’ identification was not documented in a systematic manner.

## 2. Neurobiological Determinants of Anorexia

From a macro perspective, anorexia leads to a decrease in brain volume [25]. This applies to both gray and white matter, with a reduced number of astrocytes and no change in the number of neurons or oligodendrocytes [26]. As those alterations resolve after weight recovery, they seem to be a combined effect of malnutrition and dehydration. On the other hand, a micro perspective gives a deeper insight into the neurohormonal pathways that may underlie the development of anorexia nervosa. Neurochemical concepts of the anorexic state include two major theoretical currents that revolve around dysfunction in the reward system and/or appetite regulating neuropeptides.

### 2.1. Opioids as Key Regulators of Dopaminergic Activity

The hypothesis regarding the importance of endogenous opioids in the ingestive behaviors has arisen from studies describing the reduction of short-term food intake in subjects with normal body weight after the administration of general opioid antagonists [27]. Later studies showed that opioids should play the most accentuated role in the hedonic assessment of food, which allowed to construct the hypothesis of opioid palatability assuming that eating tasty food increases the level of endogenous opioids and stimulate further eating. In addition to its role in food-related behaviors, the opioid system also protects from starvation by reducing metabolism and preserving energy.

Both food and physical activity are known for their ability to activate reward pathways involving the mesolimbic (from ventral tegmental area to nucleus accumbens) and nigrostriatal (from substantia nigra to dorsal striatum) dopamine systems. Anorectic patients present with anhedonia, food aversion, and excessive physical activity indicating abnormal reward processing that might be explained by dysfunctional dopamine system. Moreover, an extreme degree of starvation and physical activity despite the lethal consequences along with the denial of the disease and the high risk of relapse resemble the mechanisms observed in addiction. Such observations have increased interests in the role of endogenous opioids, which are known to interact with the dopamine reward circuits, and formed the basis for reflection on the role of endogenous opioids in the pathogenesis of anorexia nervosa [28]. Analysis of the cerebrospinal fluid (CSF) revealed the increased total opioid activity among emaciated patients with anorexia nervosa [29]. However, further in vivo analyzes of individual endogenous opioid molecules have given inconclusive results, and most notably reports dates back to the 1990s. (Table 1). Dynorphin levels in the cerebrospinal fluid were normal at all stages of anorexia nervosa, while β-endorphin (β-EP) levels were shown to be normal or decreased with a tendency to normalize after weight restoration [30,31]. Since β-EP has been shown to be responsible for the satisfactory (hedonic) properties of food consumption, it has been hypothesized that its reduced central concentration may be the cause of reluctance to eat observed in AN [32].

On the other hand, most measurements of peripheral opioids (mainly β-endorphin) have presented an increase in their concentrations [36,37,40,41]. In addition, the dynamic secretion of peptides derived from pro-opiomelanocortin (POMC)—β-lipotropin (β-LP) and β-endorphin—seems to be altered in the AN population. Dynamic peripheral secretion of both β-LP and β-EP has been shown to be increased, but β-EP levels were significantly elevated only in the early night hours, while β-LP was overproduced both during the day and at night. If we consider that both peptides originate from the common precursor (POMC) and β-EP is a product of β-LP transformation, changes in the dynamic peripheral secretion may indicate the dissociation of these peptides understood as they represent different secretory sources [39]. The fact that some of the abovementioned changes have normalized after gaining weight suggests that these disorders are more likely the consequences of malnutrition, weight loss, and changes in food behavior than being the cause of AN, and may not reflect the pattern of changes in the opioid system at the onset of the disease. However, genetic studies have shown a link between anorexia nervosa and the gene encoding the delta-opioid receptor (OPRD), making it a promising target for future experiments [42].

Opioids are key regulators of dopaminergic activity, and the breakdown in dopamine elements of the complex reward system is considered partially responsible for destructive behaviors in AN. (comprehensively reviewed by O’Hara et al., 2015—the authors analyze multiple AN models and propose their own focused around reward mechanisms [43]). The concentration of homovanillic acid (a dopamine metabolite) in the cerebrospinal fluid decreases with an increased binding of D2/D3 striatal receptors in women who have recovered from anorexia nervosa [44,45]. In these women, striatal and insular brain regions involved in generating hedonic impact were hypoactive to taste stimuli in hungry state [46,47]. In contrast, when exposed to the anticipation task, visual conditioned food stimuli caused an increased insular response [48]. Furthermore, reward prediction error studies have shown that in AN individuals, brains regions associated with dopaminergic signaling are highly activated when reward is different from its prediction [49,50]. Importantly, it seems that dysfunctional recognition of rewards is not distorted by an incorrect perception of stimuli, including taste and smell, because patients with AN do not present deficits in sensory systems [51]. Although they can identify tastes and odors similarly to the healthy subjects, it seems that an inadequate response may result from faulty communication between brain regions responsible for food-related signal perception and motivation. It has recently been shown that in AN patients who were administered sucrose orally, effective connectivity was directed from the ventral striatum to the hypothalamus, unlike the control group [50,52]. This research defends the hypothesis that motivational systems may exert an undue inhibitory effect on eating behavior. Those findings implicate an abnormality in the interoceptive system and faulty reward processing as important mechanisms of AN pathology [43,53].

Overall, it appears that anorexic patients, although generally referred to as anhedonic, demonstrate increased activity in the reward system but in response to disordered stimuli. Due to alterations in limbic dopaminergic transmission they perceive an experience evoked by stimuli associated with starvation and physical activity as highly rewarding, while being hyposensitive towards food. However, given the currently published data, no definite conclusions can be drawn as to the direction of these changes.

### 2.2. Hypothalamic Regulation

The neurobiological approach to the pathogenesis of AN cannot omit the role of the hypothalamus as the primary brain region involved in the regulation of food intake, as well as its integration with neuropeptides and peripheral hormones. A vast number of observed changes in appetite-controlling metabolites is secondary to malnutrition (Table 2), and resembles a simple starvation model. However, patients with anorexia nervosa do not adequately respond to homeostatic body signals that should stimulate weight restoration. The investigation of in vivo hypothalamic responses to nutrient ingestion showed a reduced reactivity upon glucose administration (i.e., a decreased activity of the hypothalamus following glucose infusion was not observed) [54,55]. The same study reported impaired functional connectivity between the hypothalamus and mesocorticolimbic reward system. Prediction error has been characterized by dopamine-dependent pattern of activation directed from the striatum to the hypothalamus that may override physiological feeding control [50]. It has been suggested that this pathway may be responsible for anxiety associated with food consumption [56,57].

The physiological hypothalamic reaction for starvation includes an increased expression of orexigenic hormones—neuropeptide Y (NPY) and agouti-related peptide (AgRP). The secretion of both peptides is the result of activated AgRP neurons leading to food-seeking adaptive behavioral responses (including modulation of fear and aggression) [60]. An activity of AgRP neurons strictly depends on peripheral signaling mediated by ghrelin (extensively discussed in the entero-endocrine alterations section of this review) acting through the growth hormone secretagogue receptor (GHSR). Since the ghrelin-AgRP pathway is vital for maintaining energy balance by both metabolic and behavioral adaptation, it may account for anxiety-relieving effects of food restriction as observed in fasted mice [61]. However, despite elevated ghrelin levels, which is a common observation in AN patients, and its peripheral fluctuations after glucose ingestion comparable to healthy controls, it has been shown that the hypothalamic response to glucose is blunted [56]. It has been hypothesized that ghrelin’s inability to stimulate appetite may result from an unspecified resistance which may have a genetic basis [62].

In parallel, fasting leads to a decrease of satiety hormones such as leptin, PYY and insulin. Loss of leptin signaling through its receptor on the AgRP/NPY neurons should provoke hyperphagia and reduction in energy expenditure not observed in AN [63]. However, there is some controversy regarding NPY in patients with anorexia that relates to reports of an increase as well as a decrease or no change at all in its plasma concentrations [64,65,66,67]. Plasma NPY may reflect its peripheral synthesis, nevertheless, despite postprandial reduction of ghrelin, NPY response to meal consumption seems to be blunted while exercise causes its increased secretion [66]. Importantly, the effect of NPY varies depending on the stimulation of various Y receptors. Thus, in addition to the Y1 or Y5 orexigenic receptors, activity via the Y2 receptor reduces food consumption [68]. Moreover, NPY neuronal activity is modulated by 26RFa neuropeptide and orexins expressed in various hypothalamic nuclei (orexins mainly in the lateral hypothalamic area). Plasma concentrations of these peptides are elevated in AN which is attributed to malnutrition adaptive mechanisms, but although it is known that orexins increase food intake, they also promote physical activity and heat loss [69,70]. Chronic overexpression of orexins finally may lead to a net decrease in body weight.

The hypothalamus also contains neurons that co-express anorexigenic pro-opiomelanocortin (POMC) and the peptide cocaine- and amphetamine-regulated transcript (CART). POMC, as a precursor, is processed into several short peptides, including the highly anorexigenic alpha-melanocyte-stimulating hormone (a-MSH) that suppresses food intake and increases energy expenditure [71]. Its expression in response to fasting is decreased due to a drop in leptin, insulin and glucose levels and the inhibitory activity of NPY/AgRP on melanocortin receptors. Also, POMC neurons in the brainstem were shown to mediate the suppressive effect of cholecystokinin on feeding [72]. Epigenetic testing showed no difference in POMC promoter DNA methylation between AN and healthy controls, suggesting adapting the melanocortin system to nutritional status rather than a specific feature of the disease [73]. That is why plasma α-MSH levels are reduced and have a positive correlation with the leptin concentration [67].

To further complicate consideration of neurohormonal pathways in anorexia nervosa, there is a number of newly discovered peptides considered potential candidates for a missing link in disturbed central feeding regulation. Fasting has been shown to reduce the concentration of various hypothalamic neuropeptides including kisspeptin, known for its impact on hormonal regulation of a reproductive status [74]. Kisspeptin stimulates the secretion of gonadotropin-releasing hormone (GnRH) and thus subsequently increases the serum level of luteinizing hormone (LH). Knocking out the kisspeptin receptor (KISS1R) leads to infertility and decreased energy expenditure with low locomotor activity and increased adiposity. Serum levels of kisspeptin seem not to significantly vary between anorectic and healthy patients and its association with BMI is uncertain [75,76,77,78]. However, it has been reported that subcutaneous administration of kisspeptin in a rat model can change the weight loss pattern, increase food intake, and modify the hypothalamic neurochemical profile [79]. Moreover, there is a negative correlation of kisspeptin with physical activity in human studies of anorexia [76].

Another disorder of neuropeptide signaling in AN is a reduced concentration of a peptide called phoenixin, mainly expressed in the hypothalamus, but also identified elsewhere in the brain and peripheral tissues [77,80,81]. Its receptor (GPR173) is expressed in both kisspeptin and GnRH neurons, which, when stimulated, leads to the GnRH-mediated secretion of gonadotropins. Phoenixin has been reported to increase food intake and correlate positively with BMI and also has some potentially anxiolytic effects on patients’ emotional states [77,82,83]. However, it has not yet been determined whether reduced plasma levels of phoenixin as an orexigenic factor may imply impaired appetite stimulation or it is secondary to depleted peripheral sources such as adipose tissue or gonads.

The recently discovered hypothalamic nesfatin-1 is also seen as potentially involved in the development of AN. It is believed to act as anorexigenic factor that inhibits food intake. Yet, there is a limited number of studies with contradictory results regarding its plasma level and a correlation with BMI [84,85]. However, nesfatin also appears to mediate anxiety, which often accompanies anorexia [84].

Although crucial in the development of AN, abnormalities in the feeding control system are only part of the complex neurobiological network behind the symptoms. Reviewed studies support the concept that homeostatic signals are overridden by pathological reward-related behaviors represented by alterations in interconnected brain circuits. However, a group of researchers has recently identified several loci in a genome-wide association study and suggested that a predisposition to anorexia is correlated with an increased risk of metabolic traits such as low BMI, indicating that energy imbalance may not only be secondary to psychological features of AN [86].

The hypothalamic response in AN appears to be partially normal, and most fluctuations of neuropeptides are believed to be dictated by adaptation to maintain healthy body weight, but to no avail. A wealth of research investigating the ineffectiveness of these mechanisms in stimulating appetite tends to view it in the context of overlapping factors, including genetic predisposition, pleiotropic effects of over-expressed neuropeptides on reward circuits, and antagonism of the over-regulated HPA axis. Such an integrative model does not fully explain, but tries to provide insight into the disease and its consequences, which further aggravate symptoms.

### 2.3. Neuroinflammation

Undoubtedly, chronic stress caused by numerous psychosocial pressures is an integral part of anorexia nervosa. Chronic restriction of food as physical stress activates physiological adaptive mechanisms, such as hypercortisolemia (along with CRH and ACTH), which normalizes when body weight is regained. As CRH itself during stress presents an anorexigenic effect, its hypersecretion in AN potentiates the disease. Hyperghrelinemia additionally aggravates these changes [87]. Apart from maintaining a sufficient glycemia with antagonistic activity to leptin and insulin, cortisol has an immunomodulating effect. As chronic stress is known to affect the immune system, various stressors in anorexia nervosa may evoke a systemic low-grade inflammation. The glucocorticoid feedback regulation in the inflammatory immune response, which in the case of hypercortisolemia would down-compensate for pro-inflammatory mechanisms, appears to be impaired in AN [88]. Indeed, the production of tumor necrosis factor-alfa (TNF-α), IL-15, and IL-6 has been reported to increase [89,90]. Those pro-inflammatory cytokines present anorexigenic effect. IL-6 reduces food intake and gastric emptying while chronic TNF-α exposure causes cachexia characterized by anorexia, weight loss, and depletion of whole-body protein and lipid [91]. Proinflammatory cytokines were shown to activate anorexigenic POMC neurons as well as inhibit AgRP secretion and NPY signaling [92]. Plasma concentrations of TNF-α and its soluble receptor were significantly higher in anorectic patients than in controls, and these remained altered even after weight restoration [93,94]. There is evidence that systemic inflammation might be linked to anxiety and depression, functional gastrointestinal disorders, as well as for inflammatory bowel disorders [95]. This may suggest that the hypothalamic inflammation and degeneration seen in anorectic mice (*anx*/*anx*) is also characteristic for AN patients [96,97].

Presented data provide the evidence for neuroinflammation as another component of the chain reaction that drives disease-related behaviors. High intensity of psycho-emotional, as well as, physical stress triggers a systemic inflammatory reaction involving the structures of the central nervous system. Released stress hormones and pro-inflammatory cytokines seem to enhance self-induced starvation through anorexigenic effects on the feeding control system, thereby exacerbating symptoms to the life-threatening level.

## 3. Autonomic Nervous System

Changes associated with anorexia nervosa affect both the sympathetic (SNS) and parasympathetic (PNS) branches of the autonomic nervous system (ANS). The most commonly described pathology is the relative predominance of PNS over SNS, resulting in clinical symptoms including bradycardia, hypotension, orthostatic hypotension, and hypothermia [14,98,99,100,101].

SNS dysfunction in patients with anorexia manifests itself both in lower basal levels of noradrenaline (NA), as well as a reduced noradrenaline release in response to normalized exercise, orthostatic stress, or feeding [102,103]. When an exercise-related increase of norepinephrine is suppressed, this results in significantly lower maximum systolic blood pressure, maximum heart rate, and maximum oxygen consumption, which generally reduces tolerance and adaptation to physical activity [104]. Interestingly, a reduced adrenergic response persists in anorexic patients with a restored body weight even a year after treatment. In addition, Lechin et al. investigated the changes within the branches of the sympathetic nervous system (adrenal sympathetic branch vs. neural sympathetic branch) and observed a significantly lower exercise-related ratio of noradrenaline: Adrenaline in the anorectic group compared to the control group [105,106]. This observation was interpreted as a consequence of the adrenal dominance over the neural sympathetic part of SNS (dissociation of the sympathetic nervous system). It seems to confirm that the deficiency of neural sympathetic function cannot be fully compensated by the sympathetic activity of the adrenal glands. This change is induced by the arousal of C1 neurons residing in rostral ventrolateral medulla with parallel inhibition of A6 and A5 neurons in locus coeruleus. Activation of C1 causes excessive stimulation of the SNS adrenal branch, while inhibition of A5 neurons limits the neuronal part of SNS. This partial and insufficient activation of SNS may result in relative parasympathetic hyperactivity, manifested by the autonomic symptoms of anorexia nervosa, but further research is needed to confirm this pathophysiological mechanism. Notably, the noradrenergic system via its projections to the arcuate nucleus can markedly impact feeding behaviors. Noradrenaline exhibits significant orexigenic activity in hypothalamic neurons increasing NPY/AgRP and reducing POMC activity via separate receptors [107].

In conclusion, patients with AN show a decrease in sympathetic activity and possibly over-activation of the parasympathetic system, which together result in a range of clinically important symptoms and potentially severe cardiac complications. These changes appear to be a consequence rather than a cause of weight loss, with the aim of reducing energy expenditure.

## 4. Gut–Brain Axis Dysregulation

Submucosal and myenteric plexuses of the enteric nervous system (ENS) regulate peristaltic activity, water, and electrolyte secretion together with local blood flow in the small and large intestine. In the stomach, intrinsic components of the gut–brain axis are underdeveloped and the organ is controlled by brain stem (medulla oblongata), although neurons projecting from the brain stem act via the ENS. The peristaltic activity of the stomach relies, independently of nerve activity, on the rhythm of the slow waves of the muscle [108]. The vagus nerve and non-vagal splanchnic mesenteric nerves form the peripheral extrinsic component of the gut–brain axis [109], and primarily project to the nucleus of the solitary tract localized in the brainstem [95]. The stimulation of mechano-sensitive vagal or spinal afferent fibers through distension of the gastric wall is responsible for the perception of fullness, and consequently controls the meal size [95,110,111,112,113,114,115]. Additionally, macronutrients influence motility and appetite regulating molecules, and further affect energy intake, but do not reach consciousness [114]. Dysregulation of these complex physiological processes negatively affects eating behaviors, and might provoke the most frequent gastrointestinal symptoms observed in AN (further discussed in the following sections), including excessive postprandial fullness, bloating, nausea, vomiting, or regurgitation) [115,116]. Microbiome dysbiosis and microbial metabolites as well as entero-endocrine alterations (also further discussed) contribute, as major etiological factors, to the gut–brain dysregulation and negatively impact satiation and satiety in AN. Recent scientific reports have confirmed the particular importance of gut heath in AN through the short- and long-term nutritional rehabilitation.

### 4.1. Microbiome Dysbiosis

A gut microbial imbalance in AN patients may affect mood, behavior, and appetite, influence weight gain and host adiposity, disturb the development of the gut mucosal immunity, and further deregulate the hypothalamic–pituitary–adrenal axis, and those relationships are undoubtedly bi-directional [117]. Short- and long-term dietary changes immensely influence both the composition and the abundance of the gut microbiota [118]. In AN, the starvation effects are similar, but are not identical to those observed in famine. The weight loss maintenance is constantly on the patients’ mind and their knowledge about the nutritional value of food determines the meals’ quantity and quality [119]. An altered diet reshapes AN patients gut microbiota, which might exacerbate or perpetuate the disease by modulating intestinal homeostasis, eating behaviors, mood, and finally weight loss [120]. Bacterial transplantation from children with kwashiorkor, a severe acute form of malnutrition, into germ-free mice fed with a low caloric density and nutrient-deficient diet resulted in a much greater weight loss in recipient mice than those transplanted with the microbiota of healthy animals [121]. What is more, Hata et al. demonstrated that gnotobiotic mice with the gut microbiota of female anorectic patients (gAN) gained weight weakly, expressed decreases in appetite and food efficiency, and were more anxious (open-field and marble-burying tests) than the mice with the gut microbiota of healthy controls (gHC). Additionally, the brain stem serotonin levels in gAN were lower in comparison to gHC [122]. Breton et al. hypothesized that a gut dysbiosis may precede the onset of AN and trigger the start of a vicious cycle where the aberrant diet further alters the composition of the intestinal ecosystem and exacerbate the clinical symptoms of this disease [120]. So far, results regarding bacterial abundance and alpha diversity of AN patients’ microbiota are inconclusive. A reduction in microbial diversity was related to impaired immune defense and limited ability to obtain calories from food [123]. Inconsistent results (Table 3) are observed between studies regarding the Firmicutes/Bacteroidetes ratio or the identity of altered levels of bacterial taxa in these patients [122,124,125,126,127,128,129,130,131,132], either. According to Fava et al. an increased level of Bacteroidetes were linked to weight loss, which is, at least partly, characteristic for AN patients [133].

In AN patients, methanogen Methanobrevibacter smithii was found to be increased, which could be a result of an adaptive response to the efficient energy extraction from lower energy-dense diets [124,129]. This increase in M. smithii was also reported in patients suffering from constipation, specifically in patients with a constipation-predominant subtype of irritable bowel syndrome. In these patients, the rise of M. smithii was negatively correlated with the stool frequency [134]. Some evidence suggest that methane slows gastrointestinal motility and thus may contribute to constipation [120]. Another study confirmed the increased levels of M. smithii in fecal samples from individuals with BMI levels below 25 kg/m^2^ compared to those with BMI above 25 kg/m^2^ [125]. Yet, Mack et al. reported a detection of the archaeon Methanobrevibacter in less than 20% of AN patients [128]. Kleiman et al. (2015) reported changes, especially among the Ruminococcaceae family during AN renourishment, similar to those observed in inflammatory intestinal diseases [126]. Mack et al. reported a low relative abundance of Bacteroidetes and carbohydrate degrading taxa (for example, Roseburia spp. and Gemminger spp.), and a high of mucin and protein degrading taxa in AN patients in comparison to age-matched healthy women at the time of hospital admission. Bacteroides and Parabacteroides spp. were decreased after renourishment, while Firmicutes increased in comparison to age-matched healthy women. No differences for the carbohydrate utilizing Roseburia spp. and Gemmiger spp. between AN patients and age-matched healthy women were reported anymore. Ruminococcus increased, while Verrucomicrobia spp., Anaerotruncus spp. and Akkermansia spp. decreased during weight gain of AN patients [128]. And indeed, Akkermansia muciniphila levels were positively correlated with weight-loss and negatively correlated with body weight gain [135,136]. Akkermansia muciniphila, a mucin degrader, is a bacterium living within the gut mucus layer [137] and its abundance has been related to fasting [138] further like elevated fiber intake [139]. According to Borgo et al., the relative abundance of Ruminococcus, Roseburia, and Clostridium genera (Firmicutes) was decreased [128], and importantly, decreased levels of Roseburia spp. were also associated with inflammatory bowel diseases [140,141]. According to Hanachi et al. gut microbiota dysbiosis (unknown genera belonging to Peptostreptococcaceae family, Dialister, Robinsoniella, and Enterococcus) in malnourished AN patients was correlated with the severity of functional gastrointestinal disorders. Pathogenic genera (Klebsiella, Salmonella) were overrepresented while symbionts (for example, Eubacterium and Roseburia) involved in immune balance were underrepresented in AN [132]. Enterobacteriaceae were more abundant in AN in comparison with the control, and thus authors hypothesized that their higher abundance could be linked to the elevated production of neuropeptide caseinolytic protease b (ClpB), which in turn could deregulate gut–brain communication in AN patients [129]. A correlation between ClpB and α-melanocyte-stimulating hormone production was associated with satiety and anxiety in eating disorders [142]. ClpB constitutes an example of a bacteria-derived molecule linking the gut microbiota with hypothalamic circuits controlling host appetite [143,144]. Morita et al. (2015) also demonstrated that the levels of Bacteroides fragilis group in ANR and ANBP patients as well as Clostridium coccoides in the ANR patients were essentially reduced in comparison with the control group. Clostridium difficile was detected only in the ANBP patients, and the colonization might be due to ANBP-specific behaviors, such as recurrent purging [127]. Hata et al. also reported a lower relative abundance of the phylum Bacteroidetes in AN patients in comparison with healthy matched controls; however, both the long-term and short-term Bacteroides treatment (with B. vulgatus as a predominant species of the B. fragilis group) did not exert any influence on weight gain in gAN mice, but successfully reversed compulsive behavior in those animals [122]. The correlation analysis revealed that Bacteroides uniformis (another example of species from the B. fragilis group) was negatively correlated with BMI [129].

Limited data is available regarding the influence of hyperactivity in AN patients on the gut microflora, either. Excessive physical activity has been noted as a typical AN feature since the 19th-century descriptions [20], and thus patients during renourishment are to reduce their physical activity in order to reduce their energy expenditure rates and advance weight recovery. In general, physical exercise is believed to improve gut homeostasis [145]. So far, Mörkl et al. reported that physical activity was associated with an increased gut microbiota alpha-diversity [130]. Although data remains unclear whether supervised physical activity during renourishment might be healthful for anorectic patients, physical activity itself has been reported beneficial for depression, anxiety, and bone mineral density, which are all expected comorbidities of AN [146].

Collectively, these results confirm the gut microbial imbalance characteristic for AN patients, but it is too early to draw definite conclusions, especially casual links. The fact that anorexia nervosa is a heterogeneous entity only makes the matter worse. So far, most studies enrolled a very limited number of participants and variable inclusion and exclusion criteria were utilized (Table 3). The individuals menstrual cycle and the estrogen levels might have influenced on gut microbiota [131] but were not take into account in the mentioned studies. Nor were the information regarding participants’ mode of birth, and whether they were breastfed, which should have influenced their microbiome [8]. Of note, there are several limitations of those studies, such as different sample collection procedures, quantification methods of bacterial species in feces samples, selection methods of the control group, choice of time point for an investigation or patients’ diet composition (including the use of food additives) together with levels of calorie intake. It should be also kept in mind that characterized feces samples may significantly differ from the actual ecosystems present in the colon or the small intestine [120]. Longitudinal dietary intervention studies with larger and younger cohorts, and more male participants are in need to advance the research in this field.

In general, since the diet has the ability to reshape the host microbiome and promote its successful survival, which should further influence the host immunity and the central reward pathways via the gut–brain axis, the particular attention should be paid to dietary habits of the whole AN families before and after the diagnosis. And thus, it would also be advisable to screen and compare the intestinal microflora among the family members (cohabitants) of AN patients instead of the age-matched healthy controls. And further, those results should be also paired with their genetic profiles. In our opinion, such studies should advance the research regarding the unestablished (but hopeful) role of the microbiome in AN pathogenesis. Surely, its role in the AN pathobiology is certain (based on animal studies), however, since the available results are inconsistent, probably due to the abovementioned methodological discrepancies, a complete definition (pathomechanisms) of such role could not be presented.

### 4.2. Microbial Metabolites

Gut microbiota forms an individual metabolic organ with the ability to produce numerous signaling molecules, and the communication between microorganisms and their mammalian hosts is bidirectional, with either symbiotic or pathogenic relationships [117,147]. Some of those molecules can be identical to human neurotransmitters, such as, for example, gamma-aminobutyric acid produced by strains of Lactobacillus and Bifidobacterium [148], or biogenic amines such as noradrenaline, dopamine or serotonin [149], and can directly and indirectly modulate the gut–brain axis [150], and affect eating behaviors. Asano et al. reported that the majority of catecholamines detected in specific pathogen-free (SPF) mice with a normal gut microbiota were of the biologically active free type, and more than 90% of dopamine and 20 to 40% of norepinephrine detected in germ-free (GF) mice were of the glucurono-conjugated type [151]. As reported by Sudo, the total intestinal norepinephrine levels were higher in SPF in comparison with GF mice [147]. Some bacterial species have transporters such as a bacterial neurotransmitter sodium symporter family member, Leu T [152], still, it awaits further research to assess if norepinephrine and dopamine found in gut microbiota originate from bacterial production via a rate-limiting tyrosine hydroxylase-like enzyme or if they come from the gut lumen via a Leu T-like transporter [117]. Such results reveal the gut microbiota as a possible net sink for biogenic amines, for example, in order to prevent an excessive host production of neurotoxins. And indeed, some scientists have already hypothesized at the turn of the 19th and 20th century that depression or anxiety (also characteristic for AN) might have resulted from the so-called autointoxication due to those neurotoxins [153]. Until recently, the concept was largely neglected, but Sudo proposed biogenic amines to be interesting candidates of such autointoxication [147]. Biogenic amines, whether endogenous or of possible microbial origin, are engaged in the intestinal immunology which further justifies the relevance of the concept in AN studies.

Thus, some authors suggest that microbial metabolites and various signaling molecules are of more importance than the gut microorganisms themselves [154]. Interestingly, the microbial formation of short-chain fatty acids (SCFA), resulting from non-digestible carbohydrates fermentation and supplying up to 10% of the host’s daily caloric intake, not only keeps the epithelial integrity and influences immune responses but may also stimulate the secretion of satiety hormones, including ghrelin, leptin and peptide YY [147,153,154,155]. The levels of total SCFA (acetate, propionate, and butyrate) have been changeably reported in AN studies (Table 3), while branched-chain fatty acids (BCFA; especially valerate and isobutyrate), resulting from protein fermentation [156], were increased in AN patients at the time of hospital admission [124]. Yet, the casual role of both SCFA and BCFA in the onset, progression and nutritional rehabilitation in AN patients needs further investigation [157], especially due to the fact that AN patients prefer to consume relatively high amounts of fiber-rich foods.

This area of research is relatively new (analogously to other psychiatric and gastrointestinal disorders as well) and much more evidence is needed to establish either a causative or a consequential (symptomatic) role of microbial metabolites (or microbial metabolites imbalance) in the course of AN disease. The methodological approach, similarly to all microbiota-related studies, should be clearly standardized to ease the interpretation of the results, too.

### 4.3. Entero-Endocrine Alterations

Numerous appetite regulating molecules (cholecystokinin (CCK), peptide YY (PYY), ghrelin, and obestatin) are secreted by enteroendocrine cells in response to the presence of luminal nutrients. These hormones are able to act on neighboring cells (i.e., enterocytes or vagal nerve endings) as well as on distant organs such as pancreatic islets, and participate in the regulation of meal size [158]. Functional toll-like receptors (TLR 1, 2, and 4) are expressed on enteroendocrine cells and thus, these cells can simultaneously monitor intestinal immune responses to symbiotic and harmful microorganisms [159]. Thus, enteroendocrine cells maintain gut homeostasis and influence the gut–brain axis through the release of these hormones, yet their role in AN pathobiology remains inadequate and call upon well-designed studies.

The sulfated forms of cholecystokinin (CCK-8-S, -33-S, -39-S, and -58-S) bind to the A-type receptor predominantly found in the gastrointestinal tract and are responsible for the regulation of meal size and satiety, while the unsulfated tetrapetide CCK-4 binds to the CCK-B receptors predominantly found in the brain [160]. Both, increased circulating baseline CKK and postprandial CCK-8S levels were reported in AN patients [161]. A diminished CCK response was observed after a glucose load, but the body weight restoration normalized CCK levels [162]. Interestingly, it was also reported that exogenous CCK-8 was more satiating and endogenous CCK plasma levels were elevated in the fasted state as well as after a low-energy preload in older adults. And despite the elevated plasma level, older adults maintained sensitivity to the satiating effects of exogenous CCK, suggesting that enhanced endogenous CCK activity should be responsible for the anorexia of aging [163]. Yet, the most recent studies reported no difference between AN patients and healthy controls, nor any correlation between CCK and BMI. However, CCK levels at the time of hospital admission were a good predictor of gastrointestinal symptoms’ improvement. While an inability to adapt CCK levels to a lower food intake might be genetically determined in the subgroup of AN patients, and CCK levels might be also linked to neuropsychological dysfunctions characteristic for AN [164].

Peptide YY is a 36-amino acid anorexigenic hormone released by the endocrine L cells of the gastrointestinal tract as well as pancreatic endocrine cells and stomach enteric neurons, in response to food ingestion, in proportion to energy intake and meal composition. It inhibits food intake, gastrointestinal motility and secretion, and is known to modulate microbiome–gut–brain axis. The release of PYY can be stimulated by gastric acid secretion and metabolites, such as already mentioned CCK or SCFA. PYY (1–36) is cleaved enzymatically by dipeptidyl peptidase 4 to the main circulating form PYY (3–36) of more relevance regarding energy and glucose homeostasis [165]. PYY activated brain regions involved in reward processing, based on functional magnetic resonance imaging [166]. So far, heterogeneous results were reported in AN patients, and most importantly usually instead of PYY (3–36), the total PYY concentrations were investigated. It was reported that fasting and postprandial PYY and PYY (3–36) were increased in those with active disease. PYY was positively associated with a drive for thinness despite the fact that the suppression of PYY would be rather expected in AN [167,168,169]. PYY (3–36) levels were positively associated with dietary restraint and lower levels in ANBP patients (significantly lower after controlling for BMI) than in the restricting type were reported [59]. Some authors suggested an involvement of PYY in the pathogenesis of anorexia nervosa rather than an adaptive response to starvation, since postprandial PYY (3–36) levels were also increased in women with restricting type AN following nutritional rehabilitation [167]. Yet, contradictory results, decreased pre- and postprandial levels of PYY in both AN subtypes, but with no significant differences between AN subtypes, were published. And those authors proposed PYY as a marker for appetite alterations in eating disorders [170]. Recently, an additional evidence for normal basal PYY (3–36) concentrations in AN participants was provided. The longitudinal analysis in acute patients yielded no change in PYY (3–36) concentration after short-term weight rehabilitation, either. A negative correlation between PYY3–36 concentration and BMI was found at admission to treatment. And importantly, physical activity (with the ability to modulate PYY secretion) levels were controlled [171].

Acylated (and thus activated) ghrelin is thought to stimulate food intake, reduce insulin secretion and stimulate gastric motility, while desacylated ghrelin should counterbalance the orexigenic effect of acylated ghrelin. Ghrelin is mainly produced in X/A-like cells of the stomach, pancreatic and intestinal cells, and its orexigenic effects are mediated centrally via NPY- and AgRP-expressing neurons [172]. Ghrelin also activates dopamine neurons in the ventral tegmental area (VTA), increases dopamine turnover in the nucleus accumbens and stimulates food intake if locally administered to the VTA [173]. So far, results regarding ghrelin and AN patients have been consistent, suggesting a role of ghrelin in the development and/or maintenance of the disorder [172]. Fasting plasma ghrelin levels in AN patients (in ANR only) were reported to be elevated, and an increase in BMI decreased circulating ghrelin levels [174,175]. AN patients had doubled fasting and 24-h plasma ghrelin levels compared to constitutionally lean, BMI-matched and healthy, subjects. Ghrelin was increased in both AN and constitutionally thin subjects with very low BMI but different eating behaviors, suggesting that both body fat mass and nutritional status should influence ghrelin levels [176]. Interestingly, ghrelin levels positively correlated with the amount of physical activity [76]. AN subjects also displayed higher ghrelin levels compared to cancer patients with cachexia [176]. One study thoroughly examined ghrelin’s secretion in adolescent girls, which demonstrated that not only its concentration is higher in AN but also its nadir as well as total area under the curve over 12 h of nocturnal sampling were higher. Additionally, secretory burst amplitude and burst mass were elevated in AN resulting in higher pulsatile and total ghrelin secretion [87]. The decrease of ghrelin in ANBP was reinforced by the assessment of a preproghrelin-derived peptide called obestatin. The ghrelin and obestatin profiles of ANBP patients were identical with that of normal-weight subjects with bulimia nervosa, suggesting that bingeing/purging behavior should adjust homeostatic aspects of food restriction. Up-to-date the identification of the two AN subtypes solely depends on patients’ testimony, and thus the assessment of appetite-regulatory peptides has been proposed to ease differentiation between subtypes of AN [170].

The abovementioned hormones represent the most studied examples of appetite regulating molecules. So far, the results seem to be inconsistent, as one would ultimately expect an increase in orexigenic signals and a decrease of the anorexigenic ones due to starvation. However prolonged physical hyperactivity, believed to play a fundamental role in the development and maintenance of AN, activates reward pathways and might deregulate entero-endocine homeostatic mechanisms via descending pathways of the gut–brain axis, and could further cause both gastrointestinal dysfunction and aggravate neuropsychiatric instability. Still, it is too early to judge, as the methodological approaches of those studies immensely limit the interpretation and consequently perpetuate uncertainty regarding the role (either etiologic or adaptive) of these hormones in the course of the disease. Only some of the abovementioned studies recorded dietary habits or use of medications. As already stressed, the diet (and/or medications) could impact the participants’ microflora, which could affect the results by either the direct release of the entero-endocrine molecules by certain strains of bacteria or by the (in)activation of the already released ones by the host, which was not considered, either. Only one study assessed the level of AN participants’ physical activity. And while the circadian variability was taken into account for the blood collection, different diagnostic tests were used, which would make it difficult to replicate the protocols.

### 4.4. Gastrointestinal Malfunction

Anorexia nervosa frequently manifests with a range of gastrointestinal symptoms related to all parts of the digestive system and of different severity, and such symptoms can be the first physical signs of the disease. Sometimes years of unsatisfactory diagnosis and therapy, including unnecessary surgical procedures, may precede the final diagnosis of the late-onset AN [177]. Yet, it remains unknown, due to the lack of sufficient clinical data, whether these symptoms are etiologic and lead to AN, or whether they represent short and long-lasting consequences of the disorder. The presence of structural or metabolic gastrointestinal disorders in the majority of patients with eating disorders, including AN were excluded [178,179,180,181,182,183], and rather suggested an impaired gastrointestinal function [184]. It is noteworthy, a structural illness like celiac disease may increase the probability of an eating disorder development because such patients pay detailed attention to their diet, body weight, and food-related gastrointestinal symptoms that may further result in a chronic dietary restriction [185].

Dry mouth, inflammation, and erosion of the gums as well as angular cheilitis (inflammation of one or both corners of the mouth) tend to be frequent in AN patients due to reduced resting and stimulated salivary flow together with lowered pH of saliva [186,187,188] Heartburn, non-cardiac chest pain and dysphagia are frequently observed in patients with AN [189]. Damaged oral mucosa and esophageal acidic damage (alike gastroesophageal reflux disease) are typical for ANBP patients due to self-vomiting, and increase the risk of the dysplasia and cancer [190]. AN patients also reported more fullness and less hunger [191], which served as an additional argument for severe food restriction [192]. AN patients exhibited slow gastric emptying for liquids [180,193,194] and solids [178,183] in both AN subtypes [195]. Some authors (but not all [180,195]) demonstrated that gastrointestinal symptoms such as nausea, vomiting, and postprandial fullness (but not satiety [196]) correlated with slow gastric emptying [181]. Noteworthy, gastric emptying and gastrointestinal symptoms improved after weight restoration [191,194], even without reaching normal levels of BMI [197]. A meta-analysis confirmed an association between anxiety, depression, neuroticism, and functional disorders (especially irritable bowel syndrome) in AN [198]. In fact, disordered eating behaviors were increased in adult patients with irritable bowel syndrome (IBS) in comparison with non-IBS patients, ranging from 15% to 25% vs. 3% [199]. A systematic review demonstrated that approximately 23% of patients with gastrointestinal diseases had disordered eating habits, which is higher than the 10% prevalence rate reported for the general population. Studies have also shown that over 90% of patients with anorexia nervosa have functional gastrointestinal symptoms [196,200,201].

Pathophysiology of functional gastroduodenal disorders in AN is very complex, multifactorial and poorly understood due to the lack of sufficient clinical data and clinical variability among AN patients. Motor and sensory gastroduodenal dysfunction, impaired mucosal integrity, local low-grade immune activation as well as dysregulation of the microbiome–gut–brain axis signaling are all involved in etiopathogensis of functional gastrointestinal disorders [132,202,203]. According to Schalla and Stengel (2019) long-lasting food restriction together with laxative abuse led to gastric damage including autonomic nerve dysfunction, which negatively alters local neural regulation and further leads to impaired gastric accommodation, bradygastria and decreased gastric emptying [204]. AN patients presented with an increased risk of the superior mesenteric artery syndrome development, which involves vascular compression of the distal part of the duodenum and results in vomiting and abdominal pain. The compression can be alleviated through positional changes and it might be a result of sever weight loss [119]. Moreover, enhanced pain perception, visceral hypersensitivity, altered regional brain activation, infections, immune and neuroendocrine dysfunction, and genetic susceptibility have all been mentioned in regard with AN [205].

Chronic constipation negatively impacts quality of life, with a higher prevalence among females [206,207] and in AN patients might be the result of abnormal colonic function due to poor or substandard intake of food as well as electrolytic alterations secondary to laxative abuse (in ANBP) and medications (especially tricyclic antidepressants) [201,208,209]. And indeed, more than 60% of AN patients suffer from delayed colonic transit and around 40% from pelvic floor dysfunction [210]. According to Santonicola et al. (2019) the correlation between pelvic dysfunction and a history of an eating disorder (including AN) might be explained as a combination of prolonged evacuation efforts, laxative abuse, enforced vomiting together with extreme exercise, and subsequently lead to the structural damage of pelvic floor muscles in addition to atrophy and rhabdomyolysis (skeletal muscle breakdown) due to starvation [115].

It seems reasonable to hypothesize that gastrointestinal malfunction in AN should primarily result from the disturbed gut homeostasis and gut–brain axis due to numerous environmental and behavioral causes (including malnutrition and hyperactivity) combined with genetic susceptibility. Gastrointestinal malfunction and inflammation may uncover and/or aggravate the disease. Nonetheless, the etiological role of the gastrointestinal malfunction in AN remains unlike and unevidenced.

## 5. Endocrine Dysregulation

All metabolic changes in AN represent the duration and degree of body weight loss due to starvation and physical hyperactivity, and thus almost solely represent a consequence of the disease; however, their etiologic role cannot be excluded based on the available data. Once the body weight is below 60% of the normal weight, every endocrine system become affected as caloric restriction overrules physiologic adjustments [119,211]. Endocrine deregulation might exert long-lasting consequences and should be closely monitored, and the below mentioned brief examples are given to support such arguments.

Due to depleted energy reserves and to reduce its further expenditure, weight loss in anorexia is often accompanied by hypothalamic amenorrhea with inhibition of gonadotropin secretion and low estrogen levels. Although estrogen is known to inhibit food intake and seems to increase the serum leptin concentration, its deficiency in anorexia may also exaggerate anxiety [212]. Regulation of reproductive function seems to center around kisspeptin neurons that mediate metabolic signals and HPG feedback mechanisms (discussed in the hypothalamic regulation section of this review). The dysfunctional hypothalamic–pituitary–gonadal axis (HPG) with a reduced concentration of gonadal steroids not only impair the reproductive function of AN patients but also determines low bone density due to the dominance of resorption processes in bone metabolism (together with low IGF-1 levels and hypercortisolemia) [213,214]. Moreover, estrogen is involved in the normal development of neuronal pathways, such as reward circuitry, during adolescence, which is a particularly important period in the onset of anorexia [215].

Leptin, a fat-derived hormone, is assumed to initiate an adaptive response to starvation, since the reduction in leptin levels were proportional to the loss of body fat mass. Leptin acted on hypothalamic feeding circuits as well as forebrain pathways regulating taste, olfaction, learning, memory, and reward [173], and low brain leptin levels deactivated anorexigenic signals that could cause anorexia as well as activated orexigenic signals to promote eating and to suppress energy expenditure [216]. In general, leptin levels were decreased in AN [59,217,218,219,220] and increased with subsequent weight restoration [219,220]. Anorectic females with higher fat mass and higher leptin levels were more likely to have menses even so having low body weight [221]. Mean leptin levels were lower in ANBP subtype compared to restricting type but were not significant after controlling for BMI. Still, it remains unclear whether low leptin levels should be related to AN psychopathology independently of body weight [59]. Additionally, decreased bone mineral density together with low levels of bone formation markers both in healthy populations [222] and in AN patients [223] were correlated with hypoleptinemia. AN females had lower amounts of brown adipose tissue (BAT) in comparison to controls, which was associated with lower T3 levels and lower bone mineral density, but no association between BAT and BMI was found [224].

The production and secretion of growth hormone (GH) in AN was highly elevated, whereas circulating levels of IGF were low, potentiating the release of GH [87,225,226,227]. It is the effect of the acquired GH resistance that can be reversed by refeeding. There are several mechanisms responsible for GH resistance, which include reduced levels of its binding protein, decreased expression of the hepatic GH receptor, and impaired GH intracellular signaling as a result of signal transducer and activator of transcription 5 (STAT5) inhibition by fibroblast growth factor 21 (FGF21) [228,229,230]. Such observations have led to the conclusion that the malnourished organism adaptively retains energy that would be spent on IGF-mediated bone growth, and increases GH lipolytic activity that does not require IGF participation. In addition to metabolic effects, GH is considered a factor that modulates the development and function of neuronal structures, and thus affects the mental state. In particular, a recent correlation study showed that changes in the GH/IGF axis may affect the underlying psychopathology [231].

Additionally, hormones might serve as screening, diagnostic and prognostic markers, and appetite-regulatory peptides were already given as an example. However multicenter randomized controlled studies are in need to address the idea.

In conclusion, endocrine adaptations to malnutrition are responsible for the development of severe symptoms of AN, such as amenorrhea or osteoporosis, but their potentially negative impact on mental health, hindering the recovery process, should also be emphasized. Anxiety and depression may be aggravated by hypercortisolemia, hypoestrogenemia or high concentration of GH, which indicates the need for integrated treatment by specialists in various fields.

## 6. Psychopathology

A model explaining the onset and the development of anorexia nervosa is multifactorial, and these relationships are undoubtedly complex. Indeed, psychological concepts have been decidedly used to explain the disease, which according to assumptions, refer to various theoretical constructs. From a clinical perspective, results have been quite consistent in the field of personality traits analysis as predictors of food restrictions. In addition, these traits are most often analyzed in the context of characteristic personality profiles. In the classic studies of Grillo and colleagues (2003), it was found that among people with AN personality disorders with anxiety traits are twice as common (avoiding, dependent, obsessive-compulsive), and in the purging type of AN, most often borderline and histrionic personality traits are observed in comparison to control groups [232].

Contemporary research has been focused on the development of regulatory personality functions. Traits that promote the development of AN, depending on the level of organization of the personality structure, influence affective, cognitive, and social responses. Among the psychological features and mechanisms essential in the etiology of the disease are personality predispositions such as fear of adolescence, including psychosexual, related to the need to return to childhood and the desire for security; perfectionism expressed in setting high-performance standards with a strong need for control as well as carrying out tasks in accordance with social expectations; depression and low self-esteem related to a negative assessment of one’s own skills and competences, the feeling of self-ineffectiveness, which causes isolation and loneliness; difficulties in building relationships with other people based on trust and mutual exchange of positive emotions; or low interoceptive awareness resulting from a difficulty of recognizing emotional states and signals arising from self-body experience. Special importance has been given to the affective regulation, especially its manifestations, such as emotional instability, impulsiveness, anger and a tendency to self-harming behavior, in the analysis of psychological mechanisms [233,234,235].

A fairly coherent picture has emerged from the analysis of the results of socio-cultural research in various cultures indicating the importance of, above all, the standards of one’s own body image, shaped by mass media. The internalization of the value of having a slim body and dissatisfaction with one’s own body as well as socio-cultural pressure associated with the pursuit of an ideal appearance have been indicated as key to the emergence of food restrictions [236,237,238].

One of the consequences of popularizing a very slim figure, especially in women, is the pursuit of the perfect appearance, which can not only be associated with cognitive distortions related to one’s own body, but can also promote the persistence of negative affect and depression, which are predictors of eating behavior disorders. These complex interactions of socio-cultural and psychological factors justify the importance of analyzing the causes of anorexia nervosa in multifactorial models, however, they are not further discussed in the current review.

## 7. Conclusions

**Figure 1:**
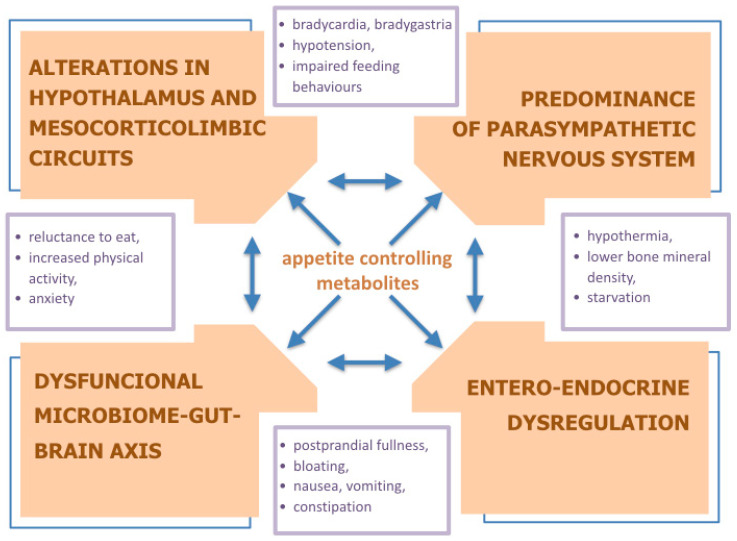
The interplay between the major pathophysiological concepts of anorexia nervosa symptomatology.

This review points the complexity of multifactorial pathogenesis of anorexia nervosa (AN), trying to concisely summarize the mechanisms involved primarily in the development of the disease itself, and secondly in the formation of the clinical picture of the patients (Figure 1). Although, it should be noted that the presented theoretical model that could fit based on the evidence reported is not evidence-based yet. Additionally, the conclusions are subject to bias due to the narrative format of the review, which certainly limits the presented results.

The importance of psychological and socio-cultural factors in the development of AN is widely established and accepted; however, there is an increasing evidence that metabolic dysregulation is much more involved than we initially thought. New concepts relate to the observed lack of reactivity to the adaptive homeostatic mechanisms activated in a state of negative energy balance, such as an increased serum concentration of ghrelin and reduced leptin or peptide YY. The neurochemical basis of these concepts revolves around the dysfunction in the reward system and appetite regulating neuropeptides, which apart from playing an adaptive role in preventing weight loss can exaggerate anxiety-like behaviors. This central anomaly could affect the microbiome–gut–brain axis and the nutritional homeostasis of anorectic patients. The autointoxication resulting from toxic microbial metabolites along with chronic stress and hormonal dysregulation tend to elicit a systemic inflammatory response, and could induce a pathological viscous cycle. Constipation was already mentioned as an anorectic symptom in the Gull’s descriptions of the disease in the 19th century; however, the scientific evidence for the importance of gut health in AN has surfaced in the last two decades. With more arguments on their way, the nutrition status and habits of the patients should no longer be considered purely in terms of caloric density. The ineffectiveness of approved treatment methods seems to result from the approach that focuses mostly on relieving the symptoms. Thus, we believe that the outlined pathophysiological components of AN may help clinicians in their therapeutic approach to thoroughly address the symptoms presented by their patients. What is more, an interdisciplinary approach should be applied both in research and clinics due to the disease heterogeneity, decreasing age of onset and increasing prevalence.

## Figures and Tables

**Table 1:** Reported variation in endogenous opioid levels in patients suffering from anorexia nervosa.

Author	Opioid or Metabolite	Location	Observation
Kaye et al., 1982 [29]	Overall opioid act.(MOR)	CSF	Increased level
Gerner et al., 1982 [33]	β-endorphin	CSF	Normal level
Kaye et al., 1987b [34]	β-endorphin	CSF	Reduced level
Lesem et al., 1991 [31]	Dynorphin	CSF	Normal level
Brambilla et al., 1985 [35]; Melchior et al. 1990 [36]; Tepper et al., 1992 [37]	β-endorphin	Plasma	Increased level
Baranowska, 1990 [38]	β-endorphin	Plasma	Reduced level
Brambilla et al., 1991 [39]	β-endorphinβ-lipotropin	Plasma	Loss of circadian rhythm (increased level) *
Brambilla et al., 1995 [40]	β-endorphin	T-lymphocytes	Increased level
Marrazzi et al., 1997 [41]	Codeine	Plasma	Increased level

* β-EP only at evening/night hours, β-LP all day, CSF cerebrospinal fluid.

**Table 2:** Changes in the concentrations of neuropeptides and hormones involved in the ingestion behaviors of anorexic patients. HPA—hypothalamic–pituitary–adrenal, CRH—Corticotropin-releasing hormone, ACTH—adrenocorticotropic hormone.

Metabolite	Concentration Shift
Neuropeptide Y, AgRP	Mixed reports
Insulin	Mixed reports
Peptide YY	Mixed reports *
Leptin [58]	Decrease
Adiponectin [58]	Increase
Nesfatin-1	Decrease
Kisspeptin	No change
Phoenixin	Decrease
Ghrelin	Increase
Orexins	Increase
26RFa	Increase
HPA axis (CRH, ACTH, cortisol)	Increase
Gonadal hormones (estrogen, testosterone)	Decrease

* Lower levels in patients suffering from the purging type than restrictive type of anorexia nervosa (AN) [59].

**Table 3:** Up-to-date studies (excluding case reports) demonstrating gut microbiota in restricting type (ANR) and purging type (ANBP) anorexia nervosa (AN).

References	Number and Sex of Participants	AN Patients Characteristics	Exclusion Criteria	Microbiota Diversity	Microbial Metabolites
Armougom et al., 2009 [124]	AN patients (*n* = 9), normal weight (*n* = 20) and obese (*n* = 20) controls, all female	19 to 36 years-old, BMI 12.73 ± 1.6 at enrollment, meeting the DSM IV-TR criteria	Use of probiotics prior to the study	Firmicutes, Bacteroidetes and Lactobacillus levels in AN patients were reported to be similar to normal weight controls.	-
Million et al., 2013 [125]	AN patients (*n* = 14 F +1 M), lean (*n* = 36 F + 40 M, overweight (*n* = 6 F + 32 M), obese (69 F + 65 M) controls	Age = 27.3 ± 10.8, BMI 13.5 at enrollment, meeting the DSM IV-TR criteria	A history of colon cancer, the presence of an inflammatory bowel disease, an acute or a chronic diarrhea 4 weeks and an antibiotic administration 6 months prior to the study	Firmicutes was found in almost all of the individuals (>98.5%), whereas Bacteroidetes was detected in 67%. Bacteroides animalis was the rarest of species (11%), and Methanobrevibacter smithii (64%) was more prevalent than *E. coli* (51%). A lower concentration of *E. coli* was found in obese vs. anorexic, lean and overweight participants, and a higher concentration of *E. coli* was associated with a lower BMI.	-
Kleiman et al., 2015 [126]	AN patients (*n* = 16; only 10 patients provided samples after weight restoration) and age-matched, healthy (*n* = 12) controls, all female	Age = 28.0 ± 11.7, BMI 16.2 ± 1.5 at enrollment, meeting the DSM IV-TR criteria, presented with less than 75% of ideal body weight	A history of gastrointestinal tract surgery (other than appendectomy or cholecystectomy), inflammatory bowel disease, irritable bowel syndrome, celiac disease or any other diagnosis that could explain chronic or recurring bowel symptoms; use of antibiotics, NSAID, steroids or probiotics 2 months prior to the study	Alpha diversity was lower in AN both before and after inpatient renourishment, however after hospital-based renourishment, intestinal microbiota diversity showed a trend toward a healthier state. Greater levels of depression were negatively correlated with the number of bacterial species.	-
Morita et al., 2015 [127]	ANR (*n* = 14) and ANBP (*n* = 11) patients and age-matched, healthy (*n* = 21) controls, all female	Age = 30.0 ± 10.2, BMI = 12.8 ± 1.3 at enrollment, meeting the DSM IV-TR criteria	Severe physical (renal failure) and infectious diseases and a history of antibiotics use or a regular intake of yoghurt or probiotics 3 months prior to the study	AN patients exhibited lower amounts of total bacteria—Clostridium coccoides group, Clostridium leptum subgroup, Bacteroides fragilis and Streptococcus in comparison to age-matched healthy women.	SCFA (acetate and propionate) levels were found to be reduced in AN patients in comparison to normal-weight participants.
Mack et al., 2016 [128]	AN patients (*n* = 55, only 44 provided samples after weight restoration), both ANR (*n* = 14) and ANBP (*n* = 11), and age-matched, healthy (*n* = 55) controls, all female	Age = 23.8 ± 6.8, BMI = 15.3 ± 1.4 at enrollment	A use of antibiotics 8 weeks prior to the study, or severe diseases including renal failure and liver dysfunction, or limited German verbal skills, or unable to understand the instructions and perform stool sampling	Alpha diversity was reduced in AN patients both before and after inpatient renourishment.	SCFA levels (excluding lowered butyrate levels) were comparable between AN patients and normal-weight participants, BCFA levels (especially valerate and isobutyrate) were increased in AN patients at the time of hospital admission and after weight gain.
Borgo et al., 2017 [129]	AN patients (*n* = 15) and age-matched, healthy (*n* = 15) controls, all female	Age not reported, BMI = 13.9 ± 2.1 at enrollment, meeting the DSM V-TR criteria	Use of antibiotics or probiotics a month prior to the study, celiac disease, irritable bowel syndrome, history of colorectal cancer, diabetes mellitus, binge eating or purging behavior, recent enteral/parenteral nutrition	An unbalanced Gram positive/Gram negative relative abundance as well as Bacteroidetes enrichment and Firmicutes depletion was characteristic for AN.	SCFA (in particular butyrate and propionate levels) levels were found to be reduced in AN patients in comparison to normal-weight participants.
Mörkl et al., 2017 [130]	AN patients, (*n* = 18) athletes (*n* = 20), normal weight (*n* = 26), overweight (*n* = 22) and obese(*n* = 20) controls, all female	Age = 22.44 ± 3.2, BMI = 15.29 ± 1.28 at enrollment, meeting ICD-10 criteria	Antibiotic or antifungal treatment 2 months prior to the study, daily or irregular intake of prebiotics or probiotics 2 months prior to the study (yoghurt and dairy products were permitted), acute or chronic diseases, or infections (including upper respiratory tract infections, fever, chronic inflammatory disorders, autoimmune disorders) 2 months prior to the study, alcohol- or drug abuse, major cognitive deficits, life-threatening conditions, history of digestive diseases, such as inflammatory bowel disease, and irritable bowel syndrome, history of gastrointestinal surgery (other than appendectomy), pregnancy, and period of breastfeeding	Microbial richness was reduced in obese woman and AN patients compared to athletes. Coriobacteriaceae was the only enriched phylotype in AN compared to other participants. Alpha-diversity was negatively correlated with depression scores.	-
Mörkl et al., 2018 [131]	AN patients (*n* = 17, including six with ANR type) and normal weight athletes (*n* = 20), normal weight (*n* = 25), overweight (*n* = 21) and obese(*n* = 19) controls, all female	Age = 21.79 ± 3.62, BMI = 15.22 ± 1.27 at enrollment, meeting ICD-10 criteria	Antibiotic or antifungal treatment 2 months prior to the study, daily or irregular intake of prebiotics or probiotics two months 2 months prior to the study (yoghurt and dairy products were permitted), regular intake of medication (except for AN patients), acute or chronic diseases or infections (including upper respiratory tract infections, fever, chronic inflammatory disorders, autoimmune disorders) 2 months prior to the study, alcohol- or drug abuse, major cognitive deficits, life-threatening conditions, history of digestive diseases, such as inflammatory bowel disease, and irritable bowel syndrome, history of gastrointestinal surgery (other than appendectomy), pregnancy, and period of breastfeeding	Zonulin levels were comparable when participants were divided according to their BMI. No difference on phylum level of gut microbiota between the high and low-zonulin group was reported. Ruminococcaceae and Faecalibacterium were more abundant in the low-zonulin. Increased levels of inflammatory markers (CRP and IL-6) were reported in the high-zonulin group.	-
Hanachi et al., 2019 [132]	ANR (*n* = 22) and ANBP (*n* = 11) patients and age-matched, healthy (*n* = 22) controls, all female	Age = 32 ± 12, BMI = 11.7 ± 1.5 at enrollment; meeting the DSM IV-TR criteria	Use of antibiotics 2 months prior hospitalization, diabetes, digestive pathology, metabolic disease, a history of obesity, inflammatory and/or autoimmune disease before the onset of AN	AN patients showed a reduced alpha-diversity compared to controls. The severity of malnutrition was negatively correlated with the Verrucomicrobiaceae and Ruminococcacea families and positively with the Clostridiales order, Turicibacteraceae and Eubacteriaceae families.	-
Hata et al., 2019 [122]	ANR patients (*n* = 10) and age-matched, healthy (*n*= 10) controls, all female	Age = 23.0 ± 3.4, BMI = 13.7 ± 0.1 at enrollment; meeting the DSM IV-TR criteria	A history of digestive diseases such as inflammatory bowel disease and irritable bowel syndrome and severe conditions (renal failure) and infectious diseases, and/or a history of antibiotic use or regular intake of yogurt or probiotics 3 months prior to the study	A lower relative abundance of Bacteroidetes was observed in AN in comparison to age-matched healthy women.	-

SCFA: short-chain fatty acids, DSM: Diagnostic and Statistical Manual of Mental Disorders.

## Abbreviations

ACTH	adrenocorticotropic hormone
AgRP	agouti-related peptide
a-MSH	alpha-melanocyte-stimulating hormone
AN	anorexia nervosa
ANBP	anorexia nervosa purging type
ANR	anorexia nervosa restricting type
ANS	autonomic nervous system
BAT	brown adipose tissue
BCFA	branched-chain fatty acids
β-EP	β-endorphin
β-LP	β-lipotropin
BMI	body mass index
CART	cocaine- and amphetamine-regulated transcript
CCK	cholecystokinin
ClpB	caseinolytic protease B
CRH	corticotropin-releasing hormone
CSF	cerebrospinal fluid
D2	dopamine D2 receptor
D3	dopamine D3 receptor
DSM	Diagnostic and Statistical Manual of Mental Disorders
*E. Coli*	*Escherichia coli*
FGF21	fibroblast growth factor 21
fMRI	functional magnetic resonance imaging
gAN	mice with the gut microbiota of patients with AN
GF	germ-free
GH	growth hormone
gHC	mice with the gut microbiota of healthy controls
GHSR	growth hormone secretagogue receptor
GIP	glucose-dependent insulinotropic peptide
GLP-1	glucagon-like peptide 1
GLP-2	glucagon-like peptide 2
GnRH	gonadotropin-releasing hormone
GUS	β-glucuronidase
HPA	hypothalamic–pituitary–adrenal (axis)
IBS	irritable bowel syndrome
IGF-1	insulin-like growth factor 1
IL-15	interleukin 15
IL-6	interleukin 6
KISS1R	kisspeptin receptor
LH	luteinizing hormone
MOR	Mu-opioid receptor
NA	noradrenaline
NPY	neuropeptide Y
OPRD	delta-opioid receptor
PNS	parasympathetic nervous system
POMC	pro-opiomelanocortin
PYY	peptide YY
SCFA	short-chain fatty acid
SNS	sympathetic nervous system
SPF	specific pathogen-free
STAT5	signal transducer and activator of transcription 5
T3	triiodothyronine
T4	thyroxine
TLR	toll-like receptors
TNF-α	tumor necrosis factor-alfa
TSH	thyroid-stimulating hormone
VTA	ventral tegmental area
